# Effect of Hidden Vector on the Speech of PRVA

**DOI:** 10.3389/fpsyg.2021.627148

**Published:** 2021-05-28

**Authors:** Tetsuya Matsui, Iori Tani, Kazuto Sasai, Yukio-Pegio Gunji

**Affiliations:** ^1^Department of Robotics, Faculty of Robotics and Design, Osaka Institute of Technology, Tokyo, Japan; ^2^Information Science and Technology Center, Kobe University, Hyogo, Japan; ^3^Department of Computer and Information Science, College of Engineering, Ibaraki University, Ibaraki, Japan; ^4^Department of Intermedia Art and Science, School of Fundamental Science and Engineering, Waseda University, Tokyo, Japan

**Keywords:** persuasion agent, conversation agent, product recommendation virtual agent, human-agent interaction, virtual agent

## Abstract

This study aimed to propose a novel method for designing a product recommendation virtual agent (PRVA) that can keep users motivated to interact with the agent. In prior papers, many methods of keeping users motivated postulated real-time and multi-modal interactions. The proposed novel method can be used in one-direction interaction. We defined the notion of the “hidden vector,” that is, information that is not mentioned by a PRVA and that the user can suppose spontaneously. We conducted an experiment to verify the hypothesis that PRVAs having a hidden vector are more effective than other PRVAs. As a result, it was shown that PRVAs having a hidden vector were perceived as being more persuasive than other PRVAs and strongly motivated the users to use the PRVAs. From these results, the proposed method was shown to be effective.

## 1. Introduction

Conversational virtual agents and robots are used in many kinds of social roles. These agents can be classified into two kinds on the basis of usage. One kind includes agents designed to quickly give exact information to users. This kind of agent includes navigation agents (Kremyzas et al., 2016) and emergency warning agents (Robinette et al., 2014). Users expect these agents to inform them quickly and exactly. The other kind includes agents that are expected to develop a topic and keep interacting with the users. This kind of agent has to expand on a topic and increase motivation of users to keep interacting with the agent. This kind of agent includes counseling agents (Schulman and Bickmore, 2009; Kimani et al., 2016) and recommendation agents (Qiu and Benbasat, 2009).

For the former kind of agent, the important factor is accuracy and perceived trustworthiness. Ways of increasing perceived trustworthiness are widely researched in the human-agent interaction (HAI) field. Demeure et al. (2011) explored important factors related to the trustworthiness of virtual agents. Verberne et al. (2013) showed that mimicry was useful for increasing the trustworthiness of virtual agents. Krämer et al. (2013) showed that the smile of a virtual agent increases the perceived trustworthiness.

For the latter kind of agent, the important factor is attracting the attention of users and motivating them to continue interacting. In particular, for counseling agents, it is important to create a rapport with users through long interactions (Gratch et al., 2007; Huang et al., 2011). This kind of agent requires design methods different from those of the former kind of agent.

To attract the attention of users and maintain interaction, many methods for designing conversational agents have been suggested. Some review studies surveyed suggested design methods for these kinds of agents (Laranjo et al., 2018; Montenegro et al., 2019; Rheu et al., 2021). These studies showed that many of the design methods had multi-modal and bidirectional interaction designs. The important problem is that these methods postulate devices and high-spec computers or servers.

To maintain interaction, many prior pieces of research have suggested multi-modal interaction designs. Zhao et al. (2014) suggested a dyadic computational model for constructing rapport. Vardoulakis et al. (2012) showed that choosing topics was an important factor in making older people to continue interacting with virtual agents. Small talk is also a useful factor for maintaining interaction (Bickmore and Cassell, 1999). Kobori et al. (2016) showed that small talk improves users' motivation in interviews. Li et al. (2017) showed the same effect for small talk. These pieces of research used multi-modal bidirectional interaction systems.

Also, bidirectional interaction designs have also been researched. Eye gaze in virtual agents has been researched as an important method for attracting users (Ruhland et al., 2015). Other non-verbal behaviors (nodding, smiling) have also been researched for creating rapport with conversational agents and robots (Huang et al., 2011; Koda et al., 2012). These previous studies showed that these non-verbal signs must be expressed depending on the users' behavior. Thus, we must use sensor devices to sense this behavior if we used these methods for conversational agents.

The costs of multi-modal and bidirectional interaction designs are generally high. These designs need devices to sense the behavior of users (for example, cameras, microphones, and pose-detection devices) and high-spec computers or servers to operate conversational agents. However, when virtual agent systems are used for online shops, these costs cannot be necessarily paid. Thus, exploring more simple and inexpensive methods for attracting the attention of users by agents and maintaining the interaction is important. However, little research has been done on such methods. We aim to propose a way of keeping users motivated to interact with an agent without a multi-modal bidirectional system.

In this research, we focused on recommendation agents. Recommendation agents are required to keep users motivated to interact. Product recommendation virtual agents (PRVAs) are recommendation agents that recommend products on websites (Qiu and Benbasat, 2009). We aimed to suggest a novel method for getting users to continue listening to the speech of a PRVA and increase the effect of the PRVA.

## 2. Hypotheses

**Figure 1:**
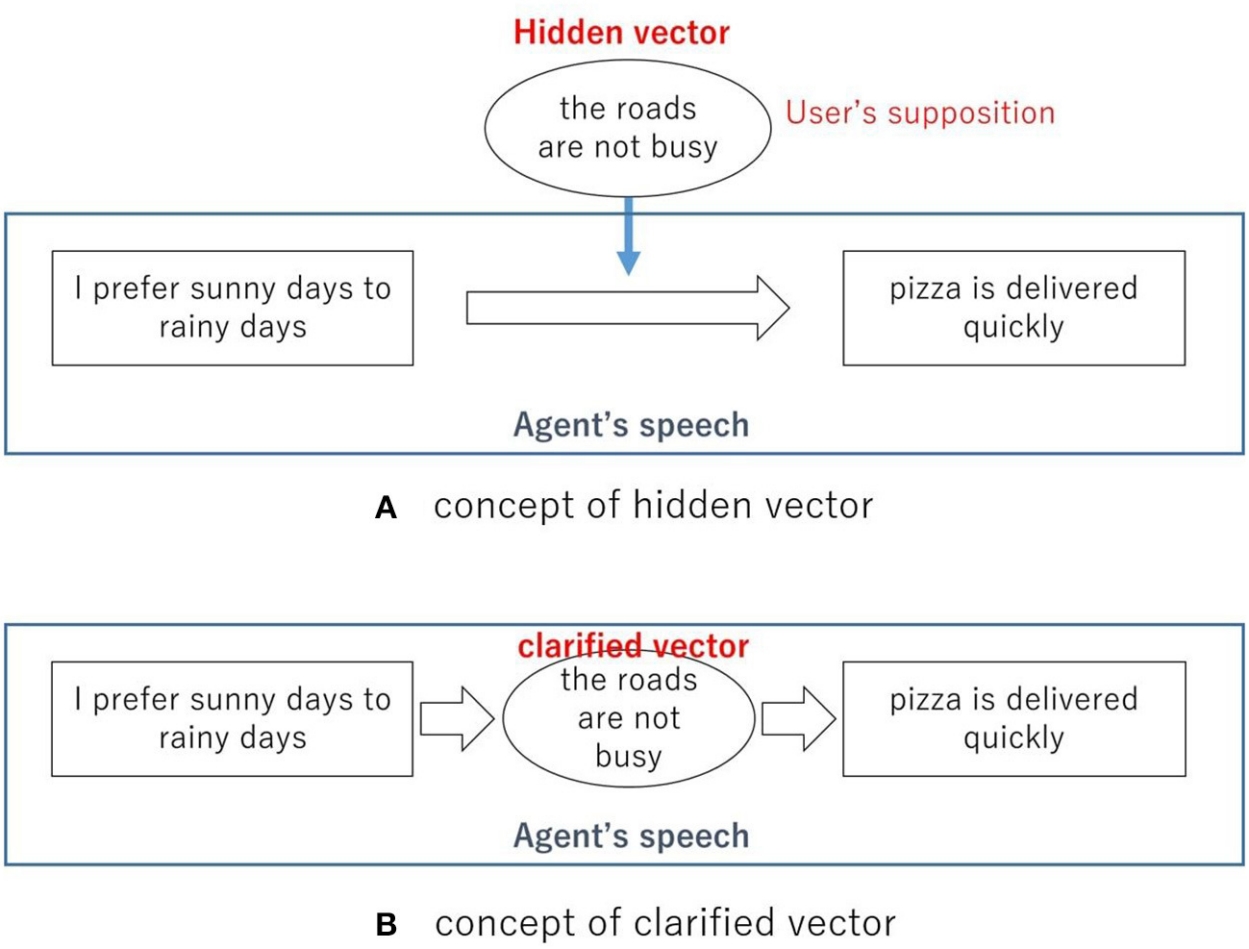
Concepts of vectors defined in this research. **(A)** Concept of hidden vector. **(B)** Concept of clarified vector.

We formulated hypotheses to suggest a method for designing conversations so as to construct an effective PRVA. In this study, we define the “hidden vector,” as information that is not mentioned in the speech of a PRVA, but users can suppose it. For example, the PRVA will say “I prefer sunny days to rainy days because pizza is delivered quickly on sunny days” in the self-disclosure phase. This is a bit meaningless because why pizza is delivered quickly on sunny days is not explained. The user can suppose the hidden reason as to why pizza is delivered quickly on sunny days, for example, “the roads are not busy on sunny days.” We define this reason that is supposed by the user as a “hidden vector.” Figure 1A shows this concept. This is different from “hidden vector state” suggested by He and Young (2005). This is not a state and this is an hidden information in interaction.

In the field of human–human interaction, the importance of hidden vector was researched in prior studies. Mower et al. (2009) showed the importance of the ambiguity of emotion expression. In some dialog models, hidden information was defined as one of the important factor (Young et al., 2007, 2010). These prior studies showed that the hidden and ambiguous information is the important factor in human–human interaction.

In this study, we hypothesized the following:

1. Users hearing the speech of a PRVA that has a hidden vector will rate the PRVA as more persuasive than users hearing the speech of a PRVA that does not have a hidden vector.
2. Users hearing the speech of a PRVA that has a hidden vector continue listening to the recommendation of the PRVA than users hearing the speech of a PRVA that does not have a hidden vector.
3. Users hearing the speech of a PRVA that has a hidden vector will want to follow the recommendation of the PRVA more than users hearing the speech of a PRVA that does not have a hidden vector.

As per our hypotheses, the hidden vector will attract the attention of users and increase their motivation to interact with a PRVA. This is because the hidden vector will increase the interest of users, for example, “Why did the PRVA say that pizza was delivered quickly on sunny days? I wish I knew the reason.” This can lead users to spontaneously interact. However, if the PRVA gives the reason themselves, users will not have a hidden vector to be curious about, so their motivation to interact will be lower. Figure 1B shows this situation. The user does not need to fill in the speech of the PRVA spontaneously, and thus, the PRVA will not attract their attention this way. We conducted an experiment to verify this.

We focused not only on the motivation of users to continue interacting but also on the persuasiveness perceived by and the recommendation effect of the PRVAs, as previous studies suggested that the persuasiveness of clerks further motivates customers to keep talking with the clerks and make purchases in e-commerce (Helander and Khalid, 2000; Kukar-Kinney et al., 2009). Thus, these three factors are intimately connected.

## 3. Experiment

### 3.1. Materials and Methods

**Figure 2:**
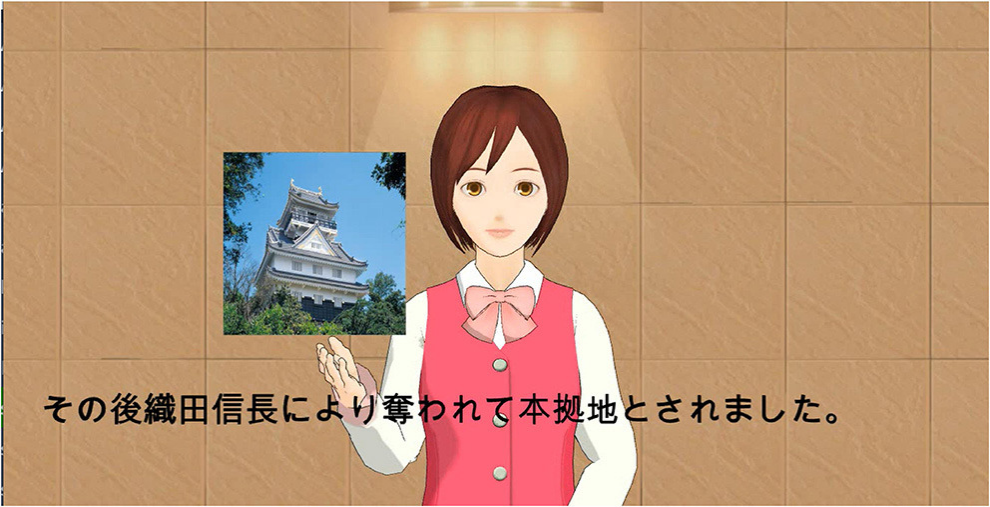
Snapshot of movie in experiment.

We conducted an experiment to verify the effect of the hidden vector. All experimental tasks are listed as follows: the participants watched movies featuring a PRVA. In the movies, the PRVA expressed self-disclosure and recommended a trip to a Japanese castle. The PRVA and the recommended trip were the same for each condition. We executed the PRVA with MMDAgent, a free toolkit for building voice interaction systems.^1^ Figure 2 shows a snapshot of the movie.

In the experiment, we verified the effect of the hidden vector on self-disclosure of the PRVA. Moon (2003) showed that self-disclosure increased rapport between humans and computers. This effect has been observed in human–virtual agent interaction (Huang et al., 2011; Zhao et al., 2014). Thus, we designed a PRVA that expressed self-disclosure before making a recommendation and executed speech containing a hidden vector within the self-disclosure.

The independent value sentences were self-disclosures given by the PRVA. In the experiment, we set three conditions.

- Condition 1: hidden vector condition

In this condition, the PRVA stated “I prefer sunny days to rainy days because pizza is delivered quickly on sunny days” in the self-disclosure phase. This sentence expressed the preference of the PRVA; however, the reason pizza was delivered quickly on sunny days was unclear. The participants were to suppose the hidden vector.

- Condition 2: clarified vector condition

In this condition, the PRVA stated “I prefer sunny days to rainy days **because the roads are not busy** and pizza is delivered quickly on sunny days” in the self-disclosure phase. This sentence expressed the preference of the PRVA, and the reason pizza is delivered quickly on sunny days was clear.

- Condition 3: control condition

In this condition, the PRVA stated “I prefer sunny days to rainy days” in the self-disclosure phase. This sentence showed only the preference of the PRVA without giving a reason. All sentences expressed in each condition are shown in Table 1. The experiment was conducted in Japanese, so the sentences were written in Japanese. In Japanese, each of the sentences in each condition had almost the same length.

**Table 1:** Sentences spoken by PRVA in each condition.

**Condition 1: hidden vector condition**
Hi, nice to meet you.
I am Mei, an online trip adviser.
Let me introduce myself.
My favorite food is pizza.
I prefer sunny days to rainy days because pizza is delivered quickly on sunny days.
How about you?
**Condition 2: clarified vector condition**
Hi, nice to meet you.
I am Mei, an online trip adviser.
Let me introduce myself.
I prefer sunny days to rainy days **because the roads are not busy** and pizza is delivered quickly on sunny days.
How about you?
**Condition 3: control condition**
Hi, nice to meet you.
I am Mei, an online trip adviser.
Let me introduce myself.
My favorite food is pizza.
In particular, I like eating seafood pizza with tabasco, fried chicken, and cola.
How about you?

The dependent values were the persuasiveness perceived, the motivation to use the PRVA, and the recommendation effect. To verify these values, we asked the participants these questions:

- Q1: How much did you feel that the PRVA was trustworthy?
- Q2: How much did you feel that the PRVA was persuasive?
- Q3: How much did you feel that the PRVA moved you?
- Q4: How much did you feel that you wanted to talk with the PRVA?
- Q5: How much did you feel that you wanted to listen to the PRVA's speech more?
- Q6: How much did you feel that the PRVA's speech interested you?
- Q7: How much did you feel that you were interested in the trip recommended by the PRVA?
- Q8: How much did you feel that you were interested in the sightseeing spot recommended by the PRVA?

Q1–Q3 were scales for measuring the persuasiveness perceived. We defined the average of the answers to these three questions as the “persuasiveness perceived” in this paper. Q4–Q6 were scales for measuring the motivation to use the PRVA. We defined the average of the answers to these three questions as the “motivation to use.” Q7 and Q8 were scales for measuring the recommendation effect. We defined the average of the answers to these two questions as the “recommendation effect.” In the experiment, we calculated Cronbach's coefficient α for each of the scales to verify them (the results are shown in the Results section). The participants answered all questions on a seven-point Likert scale.

The experiment was conducted on the web. All participants were recruited *via* Yahoo! Crowdsourcing^2^ and received 30 yen (about 28 cents) as a reward.

For condition 1, we recruited 91 participants; there were 68 males, 22 females, and 1 other ranging from 20 to 64 years with an average of 43.4 (*SD* = 9.0). For condition 2, we recruited 91 participants; there were 69 males and 22 females ranging from 23 to 76 years with an average of 47.2 (*SD* = 10.4). For condition 3, we recruited 91 participants; there were 60 males and 31 females ranging from 18 to 73 years with an average of 45.7 (*SD* = 9.9). All experiments were conducted in compliance with the ethics committee of Seikei University and Japanese law.

## 4. Results

First, we calculated Cronbach's coefficient α between the questions for each scale for each condition. For condition 1, the α between the three questions for “persuasiveness perceived” was 0.87, that between the three questions for “motivation to use” was 0.93, and that between the two questions for “recommendation effect” was 0.95. For condition 2, the α between the three questions for “persuasiveness perceived” was 0.87, that between the three questions for “motivation to use” was 0.92, and that between the two questions for “recommendation effect” was 0.94. For condition 3, the α between the three questions for “persuasiveness perceived” was 0.89, that between the three questions for “motivation to use” was 0.95, and that between the two questions for “recommendation effect” was 0.95. For every condition, Cronbach's coefficient α was more than 0.80. This means that the credibility for each scale in each condition was high.

After that, we conducted a one-way ANOVA for each scale for each condition since we conducted the experiment with three conditions. Table 2 shows the results.

**Table 2:** Averages (SDs) for each scale in experiment.

**Scale**	**Condition 1**	**Condition 2**	**Condition 3**	***F***	***p***
	**hidden**	**clarified**	**control**		
Persuasiveness perceived	3.90 (1.18)	3.47 (1.13)	3.56 (1.07)	3.03	0.025
Motivation to use	4.03 (1.41)	3.62 (1.44)	3.37 (1.40)	3.03	0.007
Recommendation effect	3.74 (1.37)	3.51 (1.46)	3.40 (1.30)	3.03	0.251

For “persuasiveness perceived,” there was a significant difference (*p* < 0.05). We conducted a sub-effect test with the Holm's method. As a result, between condition 1 and 2: *t*_(90)_ = 2.64, *p* = 0.01, between condition 1 and 3: *t*_(90)_ = 2.04, *p* = 0.04, and between condition 2 and 3: *t*_(90)_ = −0.51, *p* = 0.67.

There was a significant difference between conditions 1 and 2 (*p* < 0.05) and between conditions 1 and 3 (*p* < 0.05).

For “motivation to use,” there was a significant difference (*p* < 0.01). As a result, between condition 1 and 2: *t*_(90)_ = 1.91, *p* = 0.05, between condition 1 and 3: *t*_(90)_ = 3.16, *p* = 0.00, and between condition 2 and 3: *t*_(90)_ = 1.11, *p* = 0.35.

We conducted a multiple comparison with the Holm's method. There was a significant difference between conditions 1 and 2 (*p* < 0.05) and between conditions 1 and 3 (*p* < 0.01).

For “recommendation effect,” there was no significant difference by the one-way ANOVA. We conducted a multiple comparison with the Holm's method. As a result, between condition 1 and 2: *t*_(90)_ = 1.07, *p* = 0.43, between condition 1 and 3: *t*_(90)_ = 1.69, *p* = 0.14, and between condition 2 and 3: *t*_(90)_ = 0.53, *p* = 0.89.

## 5. Discussion

We verified the three hypotheses in section 2 from the results. For “persuasiveness perceived,” the PRVA in condition 1 was significantly higher than the PRVA in conditions 2 and 3. The significant difference between conditions 1 and 2 shows that the hidden vector was more effective than the clarified vector. Also, the significant difference between conditions 1 and 3 demonstrates the positive effect of the hidden vector. That there were no significant differences between conditions 2 and 3 showed that the topic of weather itself did not have an effect for “persuasiveness perceived,” which suggests a positive effect for the hidden vector for “persuasiveness perceived” and supports hypothesis 1.

For “motivation to use,” the PRVA with the hidden vector was significantly higher than the control agent. The PRVA in condition 1 was significantly higher than the PRVA in conditions 2 and 3. The significant difference between conditions 1 and 2 shows that the hidden vector was more effective than the clarified vector the same as “persuasiveness perceived.” Also, the significant difference between conditions 1 and 3 demonstrates the positive effect of the hidden vector. That there were no significant differences between conditions 2 and 3 showed that the topic of weather itself did not have an effect for “motivation to use,” which suggests a positive effect for the hidden vector for “motivation to use” and supports hypothesis 2.

For “recommendation effect,” there was no significant difference. Thus, hypothesis 3 was not supported by this experiment. The reason for this result seems to be the execution of the hidden vector only during self-disclosure and not during the recommendation speech. If we had executed the hidden vector during the recommendation speech, we may have been able to observe a positive effect for “recommendation effect.”

Thus, the results of the experiment support hypotheses 1 and 2 but not hypothesis 3. The results also show that the hidden vector could increase the persuasiveness perceived by the PRVA and the motivation of users to use the PRVA. The PRVA with the hidden vector was significantly higher in terms of persuasiveness perceived and motivation than the PRVA with the clarified vector. Regarding the recommendation effect, we were not able to observe a significant difference. This was a limitation of this experiment.

These results suggest a new finding for designing recommendation virtual agents. In previous studies, the virtual agents needed to interact with users in real time to keep the users motivated to interact (Bickmore and Cassell, 1999; Zhao et al., 2014; Kobori et al., 2016). However, the novel method can be executed in one-direction interaction. It can also be executed with a very simple system.

However, the experiment seemed to be limited by context. We used only one type of text and one recommendation situation. The generality of the model is our future work.

## 6. Conclusion

We developed a novel method for designing PRVAs that attract the attention of users and motivate them to interact. We introduced the notion of the “hidden vector,” this is, information that is not mentioned by a PRVA and that users can suppose spontaneously. We formulated three hypotheses regarding the effect of a hidden vector and conducted an experiment to verify them. In the experiment, we used three kinds of PRVA: one having a hidden vector, one having a clarified vector, and a control. We conducted the experiment with these PRVA conditions to measure the impression of the participants on PRVAs. As a result, the PRVA having the hidden vector was perceived as being more persuasive than the other PRVAs. Also, the PRVA having the hidden vector strongly motivated the users to use this PRVA than those having the other conditions. These results suggest a method for constructing virtual agents that keep users motivated to interact.

## Data Availability Statement

The original contributions presented in the study are included in the article/Supplementary Material; further inquiries can be directed to the corresponding author/s.

## Ethics Statement

The studies involving human participants were reviewed and approved by Ethics committee of Seikei University. Written informed consent for participation was not required for this study in accordance with the national legislation and the institutional requirements.

## Author Contributions

TM conducted the experiment and analysis, and drafted the manuscript with important contributions from IT, KS, and Y-PG. All authors participated in the review and revision of the manuscript, and have approved the final manuscript to be published.

## Conflict of Interest

The authors declare that the research was conducted in the absence of any commercial or financial relationships that could be construed as a potential conflict of interest.

## Footnotes

^1^http://www.mmdagent.jp/

^2^https://crowdsourcing.yahoo.co.jp/
